# Conduction System Pacing in Pediatrics and Congenital Heart Disease, a Single Center Series of 24 Patients

**DOI:** 10.1007/s00246-022-02942-9

**Published:** 2022-06-09

**Authors:** Amanda Gordon, Erick Jimenez, Daniel Cortez

**Affiliations:** 1 Department of Pediatric Cardiology, University of Minnesota, 5th Floor East Building, 2450 Riverside Avenue, Minneapolis, MN 55454 USA; 2 Department of Pediatric Cardiology, Cincinnati Children’s Hospital, Cincinnati, USA; 3 Department of Pediatric Cardiology, UC Davis Medical Center, Sacramento, USA

**Keywords:** His-bundle pacing, Pediatrics, Congenital heart disease

## Abstract

His-bundle pacing has demonstrated feasibility in numerous adult studies to reverse and prevent pacing-induced cardiomyopathy, however, is met with higher capture thresholds with deployment sheaths designed for adults with his-bundles in the typical location. To describe 24 pediatric and adult congenital patients post-physiologic pacing. Patients at the University of Minnesota Masonic Children’s Hospital with congenital complete heart block or congenital heart disease and atrioventricular block presented for pacemaker placement between November 2019 and January 2021. Twenty-four patients had attempted his-bundle placement using either Medtronic’s C315 or C308 sheaths and 3830 leads except for 3 patients who had Boston Scientific’s His system with the Shape 3 sheath and 7842 leads. Twenty-four total patients underwent physiologic pacing (23 his-bundle, 13 female, 11 male) with median age of 14 years (range 8–39 years) with median weight of 51 kg (range 21.2–81 kg) with five right-sided implants performed. Twelve patients had congenital heart disease including atrioventricular canal defects, tetralogy of Fallot, and ventricular septal defect repairs (nine patients with ventricular septal defect repairs). Twelve patients had selective His-bundle pacing (six with congenital heart disease). Median threshold to capture was 0.5 V at 0.4 ms (range 0.4 to 1.1 V at 0.4 ms), impedance 570 ohms (range 456–1140 ohms), and sensing median of 9.7 mV (range 1.5–13.8 mV if present). The median follow-up time was 610 days (range 240–760 days). No complications occurred peri-procedurally or during follow-up. His-bundle pacing is feasible in pediatric and congenital heart disease patients.

## Introduction

Pacing-induced cardiomyopathy affects adult patients and pediatric patients with conduction system and resynchronization therapies (CRT) being applied to attempt to improve it [1]. CRT with biventricular pacing has played an important role in patients affected by pacing-induced cardiomyopathy. Biventricular pacing assists patients with reduced function of the left ventricle by left-basal pacing typically, while also still using a right ventricular lead [1]. CRT has been shown to have less benefit for patients with normal QRS durations and depending on the site of activation delay, may not be the best solution for patients with congenital heart disease. Furthermore, CRT involved 2 ventricular leads (right ventricle and coronary sinus or two epicardial ventricular leads), thus His-bundle pacing with only one ventricular lead, has been an attractive method recently compared to CRT [1]. His-bundle pacing became a new and popular route to assisting those heart failure in the past decade. His-bundle pacing activates the ventricles through the His-Purkinje system which results in a more physiological pacing method as opposed to the traditional method [2]. This therapy provides an alternative to biventricular pacing for treating heart failure [3]. Furthermore, this therapy has been shown that pacing-induced cardiomyopathy can be reversed [4]. Other advantages in His-bundle pacing include lack of the complications that arise with typical CRT from the lead placement including phrenic stimulation and venous congestion [1]. Though there have been many studies done on adult patients with His-bundle pacing, studies in the pediatric community are lacking [4]. His-bundle pacing seems to be the superior option in order to improve overall ventricular function and additionally, the benefits that His-bundle pacing has on improving the conduction of the heart to a more physiologic state outweighs any other option [5]. Conduction system pacing establishes a synchronized rhythm within the ventricles which avoids the effects of right ventricular pacing. His-bundle pacing has been proven to be feasible and safe across all ages in smaller studies including our own prior retrospective study including the smallest His-bundle pacing case to date is 21.5 kg [4, 6]. This study found no procedural complications.

We present a case series of 24 pediatric and congenital heart disease patients with conduction system pacing, including mostly His-bundle pacing with short-term and mid-term follow-up at the University of Minnesota.

## Methods

After approval by the Institutional Review Board, a retrospective chart review of all cases of selective and nonselective His-bundle, and left bundle branch pacing was performed, with consent waived, due to the retrospective nature of the review. Patients were captured from the electrophysiology database from the University of Minnesota, Division of Pediatric Cardiology [7, 8]. All procedures were performed under general anesthesia with either intubation or laryngeal mask placement and without paralysis except while securing the airway. His-bundle pacing and left bundle pacing were performed by standard method [9].

As a review of our His-bundle pacing technique, we used a Livewire octopolar catheter (Abbott Medical, Abbott Park, IL) to mark the His either on by fluoroscopy or by 3-dimensional map (Ensite Precision, Abbott Park, USA) via either the right femoral vein or right axillary vein approach as our initial step during each of the procedures.

**Fig. 1:**
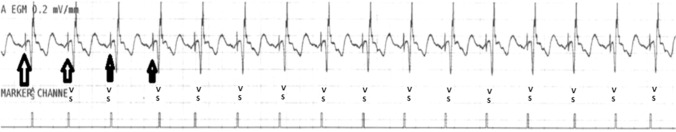
His-signal on ventricular lead after implantation

Then, a C315 guiding sheath (Medtronic, Minneapolis, USA) is inserted over an octopolar Livewire (St Jude Medical, Saint Paul, MN, USA), and positioned slightly distally to the strongest His-bundle signal on the 3D map. Subsequently, the Livewire was replaced with a 3830 ventricular pacing lead (Medtronic, Minneapolis, USA). When a His-signal was not recordable during mapping, unipolar pace mapping was used to identify an optimal site. The pacing lead is then fixed at the point where the His-signal was present and adequate sensing and threshold were obtained. Pacing at high output was performed to assess His-bundle capture prior to coiling of the lead. Atrial septal lead placement is typically our first attempted location. The rest of the procedure and attachment to the pacemaker generator is performed as per standard technique. All 3830 ventricular leads were of 69 cm in length. Atrial leads were either 49 cm or 59 cm depending on patient size. Please see Fig. 1 for the His-signal seen on the ventricular lead. We found that patients who were smaller required pre-shaping of the C315 sheath to enable a tighter bend to reach the His-bundle area. We also found the J-shape to be the best for atrial lead deployment with need to pre-split this sheath if the 49 cm lead was used.

### Data

Non-parametric data are presented as median value with total ranges reported due to low number of patients in study. A *p*-value ≤ 0.05 was considered significant.

## Results

Twenty tour total patients underwent physiologic pacing with 24 patients having His-bundle pacing (45.9% male) with a median age of 14 years (range 8–39 years) with median weight of 51 kg (range 21.2–81 kg) with five right-sided implants performed (20.8%). Twelve patients had congenital heart disease (50.0%) including atrioventricular canal defects, critical aortic stenosis, and ventricular septal defect repairs (nine patients with ventricular septal defect repairs) and one patient had myotonic dystrophy (4.2%). Twelve patients (50.0%) had selective His-bundle pacing (6 [50.0%] with congenital heart disease). At implant, the median ventricular capture threshold was 1.0 V@0.4 ms (range 0.4–1.5 V@0.4–0.5 ms, *p*-value < 0.001), impedance 570 ohms (range 437–820 ohms), and sensing median of 9.7 mV (range 1.8–19.3 mV if present). For non-selective His-bundle implants, the median His-capture threshold was 2 V@0.4 ms (range 1–6 V@0.4–0.5 ms). The median baseline QRS duration was 110 ms (range 78–190 ms) with subsequent paced QRS duration was 100 ms (range 80–140 ms). Six patients with prior pacemakers had a QRSd median of 153 ms decreased to 100 ms (*p*-value 0.001). Three patients (12.5%) had the device placed with the Boston Scientific His-bundle pacing system (# 18, 19 and 23). No complications occurred peri-procedurally or during follow-up. Please see Table 1 for full details.

**Table 1:** Demographic data for twenty-four His-bundle pacing patients in pediatric and congenital heart disease patients

#	Age (years)	Weight (kg)	Sex	Cardiac diagnosis	Access	Pacing type	Paced QRSd (ms)	R-wave (mV)	Imp (Ω)	Threshold V@0.4 ms	His V@0.4 ms	FU days	% V pacing	R-wave (mV)	Imp (Ω)	Threshold V@0.4 m	His V@0.4 ms
1	15	56	F	AVSD. SND. High grade AVB	L	NS	120	13.8	820	0.4^	2	753	0.3	14.2	830	0.5	2
2	9	29.3	F	CCHB	L	S	90	1.5	600	0.4^		760	100	N/A	418	2	
3	8	21.5	F	CCHB	L	S	75	4	551	0.5		743	100	4.5	399	1.5	
4	13	50.3	M	CCHB	L	S with LBBB	120	17.4	570	0.5		736	99.7	4.3	418	1.25	
5	13	50.6	M	Crit AS. AoVR SCHB	R	NS	100	15.3	513	0.5	2.5	695	100	N/A	380	0.75	2.5
6	9	30	M	CHB, Influenza B	L	NS	90	3.5	570	0.5		695	100	N/A	420	1	2
7	18	60	F	Heterotaxy, LAI, SND, Int IVC. A flutter, CCHB	R	LBBp	100	19.3	855	0.5		681	0.3	18.6	513	0.75	
8	39	75.8	M	d-TGA. Atrial switch. SND. Mobitz II	L	NS	80	4.8	950	0.5	1	676	0.1	6.5	437	1.125	1.5
9	9	22.1	M	Perimemb VSD. SCHB. EPM lead fracture	R	NS	120	5.25	817	0.5	2	669	100	N/A	551	0.75	2
10	14	81	M	Perimemb VSD. Surgical CHB. EPM Pacemaker induced CM	L	S with LBBB	140	13	805	1.1		632	100	N/A	399	1.125	
11	14	42.8	F	SND. Int CHB unknown etiology	L	NS	120	11.1	551	0.5	2	624	100	12.8	494	1.375	2
12	9	51	F	AVSD, CHB, MVR, EPM	L	S	80	5.6	827	0.75	1	623	100	11.5	361	1.25	
13	15	61	M	CCHB	L	S	80	3.3	551	0.75	0.75	597	100	3.625	303	1.5	
14	33	55.8	F	AVSD. Surgical CHB. MV replacement. EPM fracture	L	S	110	9	589	0.5	0.5	563	100	7.875	456	0.75	
15	16	71.7	M	BAV, Progressive intermittent CHB	L	NS	90	20	570	0.75	2	394	0.4	20	437	1	2
16	19	72.6	M	CCHB	L	S	86	13.1	472	0.8		387	100	N/A	361	0.6	
17	10	25.7	F	Progressive intermit CHB. NSVT	L	NS	80	11.3	551	0.5		366	12.6	5.875	283	2	6
18	22	43	F	Multiple VSD. SCHB. EPM. PM induced CM	L	NS	100	8.9	620	0.6^	6	367	100	N/A	380	2.7	
19	19	50.2	M	Myotonic dystrophy. intermittent 2:1 AVB	L	S	100	1.75	456	1		337	100	1.75	456	1.5	
20	8	21.2	F	Mobitz II. Intermit CHB	R	NS	99	11.4	684	0.75	4	330	100	12.6	570	1.5	6
21	27	73.3	F	CHB, AVCD	L	S	80	3	437	1		319	99.9	3.3	399	1.5	
22	11	50.8	F	SND, intermittent CHB	L	S	100	2	437	1.5		309	100	1.8	304	0.75	
23	17	49	F	CHB	L	NS	120	5.4	538	0.8^	2.8	302	100	N/A	517	1.7	2.8
24	10	55	M	CHB, VSD, EPM fracture	L	S	92	N/A	798	0.5		240	94	N/A	620	0.75	

*AVB* atrioventricular block, *AVCD* atrioventricular canal defect, *CHB* complete heart block, *CCHB* congenital heart block, *crit AS*  critical aortic stenosis, *EPM* epicardial pacemaker, *F* female, *L* left, *LBBp* left bundle branch pacing, *M* male, *MV* mitral valve, *NS* non-selective, *R* right, *RV* right, *S* selective, *SND* sinus node dysfunction

^at 0.5 ms pulse width

### Follow-up

The median follow-up time was 610 days (range 240–760 days). The median ventricular capture threshold was 1.0 V@0.4 ms (range 0.5–2.7 V@0.4–0.5 ms), impedance ohms 419 (range 283–830 ohms) with five patients programmed in a unipolar ventricular pacing mode, also with sensing median of 7 mV (range 1.75–20.0 mV if present). For non-selective His-bundle implants, the median His-capture threshold was 2 V@0.4 ms (range 1.5–6 V@0.4–0.5 ms). The median predicted longevity of devices for all patients with over 99% RV pacing (including years already functional) was 10.5 years (range 5.5–14.5 years).

### Improving Cardiomyopathy

All patients with normal baseline left ventricular function had normal left ventricular function by last follow-up echocardiogram (all patients received yearly echocardiograms). Most patients had normal ejection fractions prior to His-bundle placement and all of those patients continued to have normal biventricular function subsequently. Four patients, however, had depressed ejection fractions ranging from 32 to 45%. All four patients had improvement of at least 5–10% in their ejection fraction noted perioperatively, and these four maintained this improvement in subsequent echocardiograms.

### Patients With Posterior-Inferior His-Location Versus Those Without Altered Conduction

Nine patients had posterior-inferior His-bundle location, while 15 patients had normal His-bundle locations, however, with patient eight having a Senning repair, thus His-bundle implant was performed from the left ventricle (subpulmonic ventricle). When compared with patients with normal His-bundle locations, patients with a posterior-inferior His-bundle had a median age of 15 years (range 9–33 years) compared to 13 years (range 8–39 years, *p*-value 0.342), median 55.8 kg (43–80 kg) compared to 50.2 kg (21.2–75.8 kg, *p*-value 0.360), with 33% being male compared to 53% males for posterior-inferior His-bundle compared to normal His-position patients, respectively. The posterior-inferior His-patients had a baseline median QRSd of 145 ms (range 83–190 ms) compared to median QRSd of 90 ms (range 78–150 ms, *p*-value < 0.001), with post-pacing QRSd noted to be median of 100 ms (80–140 ms) compared to median post-pacing QRSd noted to be a median of 90 ms (75–120 ms, *p*-value 0.246) compared to normal His-position patients, respectively. Five out of nine patients (56%) with posterior-inferior His-bundles at baseline had selective His-bundle pacing, while seven out of fifteen patients with normal His-locations (47%) had selective His-bundle pacing (*p*-value 1.000). Median thresholds at baseline and at last-follow-up were 0.5 V@0.4 ms and 0.75 V@0.4 ms for posterior-inferior His-patients, while baseline and last follow-up ventricular threshold medians were 0.5 V@0.4 ms and 1.375 V@0.4 ms, respectively, for patients with normal His-bundle locations (*p*-values of 0.980–0.475 between groups, respectively). Median ventricular impedances at baseline and at last-follow-up were 805 ohms and 456 ohms posterior-inferior His-patients, while baseline and last follow-up ventricular impedance medians were 551 ohms and 456 ohms, respectively, for patients with normal His-bundle locations (*p*-values of 0.015–0.135 between groups, respectively). Median *R*-waves at baseline and at last-follow-up were 9.0–11.5 mV for posterior-inferior His-patients, while baseline and last follow-up ventricular impedance medians were 5.4–5.2 mV, respectively, for patients with normal His-bundle locations (*p*-values of 0.596–0.230 between groups, respectively).

## Discussion

Twenty-four patients underwent attempted His-bundle pacing (23 with His and 1 with left bundle pacing) with a median follow-up time of 610 days and follow-up as long as 760 days. There were no complications found during the procedure as well as during follow-up. Four patients had improvement in their ventricular function between 5 and 10% with transition to His-bundle pacing. This study, similar to our prior study, demonstrated feasibility of physiologic pacing in pediatric patients but now with mid-long term follow-up including up to 950 days [4]. The pulse-width used for each patient for this study stayed at 0.4–0.5 ms as opposed to adult studies where the pulse width is typically maintained at 1.0 ms due to higher His-capture thresholds. The previous study showed promising results for future research which was only reinforced with the larger cohort this current study had, moving from eight patients to twenty-four. Another case study looked at a pediatric patient with atrio‐ventricular block with His-bundle pacing and found no complications with short-term follow-up time [6]. While the largest prior study in pediatric and adult congenital heart disease was a multicenter cohort of patients with congenitally corrected transposition of the great arteries, where similar to nine of our patients, those patients had altered conduction system locations, whereas in our case the patients had deviated His-bundles which were posterior-inferior due to inlet ventricular defects, and as opposed to 3/11 successful selective His-bundle pacing case, in our population 5/9 patients with altered conduction systems were able to have selective His-bundle pacin [7]. This reiterates the benefits of His-bundle pacing with little to no complications during follow-up time. Additionally, it was found that the benefits of His-bundle pacing outweigh the benefits of current technology especially in the pediatric population given that these patients will continue to grow as they reach adulthood including narrowing of QRS duration, which has been demonstrated to be inversely proportional to ventricular functio [8]. Similar to adult studies, we found pacemaker-induced cardiomyopathy could be reversed with His-bundle pacing, however, not enough follow-up was performed to determine if we truly prevented pacemaker-induced cardiomyopathy in all of our patient [1]. The thresholds to capture of the His-bundle were also smaller in our pediatric patients, likely due to less overall distance of the His-bundle from the location of the proximal coil. Having a pacing method that is more similar to physiological methods will only benefit the lives of pediatric patients and reduce risks of pacing-induced cardiomyopathy [9].

Otherwise, between altered conduction patients (posterior-inferior His-location patients), selective versus non-selective His-bundle placements was similar and although different in impedance was present at baseline, thresholds were similar for both patient groups. Furthermore, impedance differences with higher impedances at baseline in the patients with altered His-bundle locations, likely represented change based on prior surgery, as all of these patients had prior inlet VSD closures (most were atrioventricular canal patients as well). Those same patients also had longer QRS durations, likely related to their own paced or surgically altered electrical conduction.

Otherwise, if epicardial pacing is performed, consideration of the LV apex versus mid-lateral LV wall for epicardial ventricular site would likely be beneficial, and no comparison to His-bundle pacing has been performed but would be worth assessing prospectively in future pediatric studies [10].

## Limitations

Patient size and lack of prospective arm were limiting in this study. We also have three patients with Boston Scientific leads but thus given the small number, did not have Power to assess differences between Boston Scientific or Medtronic device parameters. Collaborative studies are needed and likely will only be gained by collaborative studies in the future. Furthermore, there were still late increases in thresholds in some of our older patients, thus that still can exist as a limitation to the use of His-bundle pacing in pediatric and congenital patients. Furthermore, comfort level with His-bundle pacing and open-discussion with patients and families regarding possible higher thresholds is needed before each case when applying this technique broadly. Regarding our own patients, this discussion happened up-front, and all patients’ families were comfortable with proceeding given the risk–benefit of possible cardiomyopathy versus higher thresholds, but again, this should always be a discussion with newer techniques such as these.

## Conclusion

We present the largest case series of His-bundle pacing in patients of pediatric age or with congenital heart disease. His-bundle pacing appears feasible in these populations with good follow-up lead parameters and without complication. More studies on larger groups of patients are needed.
